# The Cedar Project: resilience in the face of HIV vulnerability within a cohort study involving young Indigenous people who use drugs in three Canadian cities

**DOI:** 10.1186/s12889-015-2417-7

**Published:** 2015-10-29

**Authors:** Margo E. Pearce, Kate A. Jongbloed, Chris G. Richardson, Earl W. Henderson, Sherri D. Pooyak, Eugenia Oviedo-Joekes, Wunuxtsin M. Christian, Martin T. Schechter, Patricia M. Spittal, For the Cedar Project Partnership

**Affiliations:** School of Population and Public Health, University of British Columbia, Columbia, Canada; Centre for Health Evaluation and Outcome Sciences, 588-1081 Burrard St., V6Z1Y6 Vancouver, BC Canada; Cree, Métis; University of Northern British Columbia, Columbia, Canada; Cree; University of Victoria; Canadian Aboriginal AIDS Network, Victoria, Canada; Splatsin te Secwepemc First Nation, Columbia, Canada

**Keywords:** Indigenous young people, Resilience, Trauma, HIV and HCV vulnerability

## Abstract

**Background:**

Indigenous scholars have long argued that it is critical for researchers to identify factors related to cultural connectedness that may protect against HIV and hepatitis C infection and buffer the effects of historical and lifetime trauma among young Indigenous peoples. To our knowledge, no previous epidemiological studies have explored the effect of historical and lifetime traumas, cultural connectedness, and risk factors on resilience among young, urban Indigenous people who use drugs.

**Methods:**

This study explored risk and protective factors associated with resilience among participants of the Cedar Project, a cohort study involving young Indigenous peoples who use illicit drugs in three cities in British Columbia, Canada. We utilized the Connor-Davidson Resilience Scale to measure resilience, the Childhood Trauma Questionnaire to measure childhood maltreatment, and the Symptom-Checklist 90-Revised to measure psychological distress among study participants. Multivariate linear mixed effects models (LME) estimated the effect of study variables on mean change in resilience scores between 2011-2012.

**Results:**

Among 191 participants, 92 % had experienced any form of childhood maltreatment, 48 % had a parent who attended residential school, and 71 % had been in foster care. The overall mean resilience score was 62.04, with no differences between the young men and women (*p* = 0.871). Adjusted factors associated with higher mean resilience scores included having grown up in a family that often/always lived by traditional culture (B = 7.70, *p* = 0.004) and had often/always spoken their traditional language at home (B = 10.52, *p* < 0.001). Currently knowing how to speak a traditional language (B = 13.06, *p* = 0.001), currently often or always living by traditional culture (B = 6.50, *p* = 0.025), and having recently sought drug/alcohol treatment (B = 4.84, *p* = 0.036) were also significantly associated with higher mean resilience scores. Adjusted factors associated with diminished mean resilience scores included severe childhood emotional neglect (B = −13.34, *p* = 0.001), smoking crack daily (B = −5.42, *p* = 0.044), having been sexual assaulted (B = −14.42, *p* = 0.041), and blackout drinking (B = −6.19, *p* = 0.027).

**Conclusions:**

Young people in this study have faced multiple complex challenges to their strength. However, cultural foundations continue to function as buffers that protect young Indigenous people from severe health outcomes, including vulnerability to HIV and HCV infection.

## Background

Indigenous scholars agree that although Indigenous civilizations are richly diverse, they have also shared common values and beliefs that facilitated healing, positive meanings, and confidence in the future [1]. Ceremonial methods for coping with stress in times of adversity enabled Indigenous peoples to process loss and grief [2]. Healing traditions were passed down intergenerationally as parents and Elders used story-telling and experiential learning to teach young people how to exercise resilience, or, find mental, physical, emotional, and spiritual wellbeing when they experienced difficulty [3]. Many Indigenous cultural practices, languages, and spiritual beliefs have survived despite 500 years of colonization in Canada. This reinforces the imperative to find alternatives to risk models of disease to identify sources of strength or resilience that may protect the health of young Indigenous people in Canada.

Colonization in Canada has included forced removal from traditional lands, genocide, and legislative measures to suppress Indigenous cultures, ceremonies, and economic development [4]. The *Gradual Civilization Act* of 1857 was one of the most damaging pieces of legislation as it initiated the church-state partnership that established the Indian Residential School System. Between 1874 and 1996, over 150,000 Indigenous children were forcibly removed and placed in residential schools. The system alienated children from their cultures, languages, and communities in an effort to Christianize and assimilate them into Canadian society [4]. Using corporal and degrading punishments, missionary teachers taught children to be ashamed of their Indigenous identity. It is estimated that more than 70 % of children in residential schools were routinely abused physically, sexually, or emotionally, in addition to being deprived of emotional or physical nurturing [4]. When former students returned to their home communities, many faced feelings of alienation resulting from having lost their connection to culture [3]. Further, residential schools severely disrupted traditional models of child rearing, and many former students unintentionally replicated the traumas they had experienced within their families and communities. Combined, these experiences prompted a cyclical effect of intergenerational trauma.

Intergenerational trauma is considered one of the most disastrous legacies of colonization and the residential school system [5]. The ongoing effects are evident within Indigenous communities that are struggling with interrelated crises of family violence, poverty, addictions, lack of traditional skills, lack of role models, and feelings of isolation. Moreover, Indigenous activists and scholars have maintained that provincial child welfare systems in Canada have perpetuated intergenerational trauma and fragmentation of Indigenous families [6]. Indigenous parents routinely face discrimination and racism within the child welfare system, and federal funding incentivizes long-term separations of Indigenous children from their families, communities, and cultures [7]. Consequently, though only 7 % of children in Canada have Indigenous ethnicity they comprise 48 % of children in the foster care system [8].

Research suggests that young Indigenous people living with unaddressed historical and lifetime traumas are more likely to use illicit drugs as a coping mechanism [9]. Further, young urban Indigenous people who use drugs in Canada experience high levels of injection drug use [10], residential transience [11], high risk sex [12], sex work [13], and sexual violence [14]. These cumulative traumas have also manifest as increased HIV and hepatitis C (HCV) vulnerability [15–19]. For example, extant literature has demonstrated that young Indigenous people who use drugs and have experienced childhood sexual abuse are twice as likely to be living with HIV infection [9], and those who had at least one parent who attended residential school are twice as likely to be living with HCV infection [20]. Taken together, these vulnerabilities have contributed substantially to the overrepresentation of Indigenous people among those living with HIV and HCV infection in Canada. Recent 2011 data indicates that Indigenous people constituted an estimated 12.2 % of all people in Canada newly diagnosed with HIV, which corresponded to an HIV incidence rate that was 3.5 times higher than among non-Indigenous people [21]. Likewise, between 2002 and 2008, the estimated incidence of HCV infection was 4.7 fold higher among Indigenous people than non-Indigenous people [19].

In this context of heightened vulnerability, Indigenous leaders have called for recognition of resilience among their young people, including acknowledging strengths-based factors that may be protective against HIV and HCV infection [22]. The most widely accepted definition of resilience in health sciences is *positive adaptation despite adversity* [23]. Resilience researchers have sought to look beyond deficit models of health to identify strength-based resources that promote wellness.

However, measures used to assess resilience are often limited because they are based on individualistic outcomes specifically valued by non-Indigenous cultures, such as self-sufficiency and self-esteem, and narrow definitions of healthy functioning, including staying in school and abstaining from substance use [24]. Moreover, resilience research has frequently failed to consider complex historical and cultural contexts when measuring resilience among marginalized youth and those outside of the dominant culture [25]. It follows that any consideration of resilience among young Indigenous people in Canada must acknowledge the historical and present-day injustices that impede resilience as well as the culturally-specific community strengths that support resilience [26].

A small but growing body of research in Canada has moved beyond individualistic, linear, and western notions of resilience to identify ways in which culture, language, and spirituality buffer adversity and create “cultural resilience” among Indigenous peoples [27]. Chandler and Lalonde’s [28] study involving 196 Indigenous bands in British Columbia (BC), demonstrated that factors associated with ‘cultural continuity’ − including self-governance, band-controlled health and education initiatives, and speaking traditional languages − were associated with lower rates of suicide among Indigenous youth. Very few studies have explored the roles that culture and resilience play in the health of young, urban Indigenous people. One study involving Indigenous young people in Winnipeg, Canada, found that those who believed it was important to participate in traditional cultural activities scored higher on an emotional competence scale and were less likely to use alcohol or be involved in crimes [29]. Further identifying sources of resilience may therefore be especially important for understanding and responding to HIV and HCV vulnerability among young urban Indigenous people who use drugs and who may be disconnected from their home communities, languages, cultures, and spirituality [27].

To our knowledge no previous epidemiological studies have explored resilience among young, urban Indigenous people who use drugs and experience vulnerability to HIV and HCV exposure within high risk environments. This study sought to investigate the relationship between resilience and a range of positive and negative factors, including cultural connectedness, help-seeking, historical and lifetime trauma, drug- and sex-related risk, and psychological distress, among young Indigenous people who use drugs in British Columbia (BC), Canada.

## Methods

### Study design

The Cedar Project methods have been described in detail elsewhere [15]. In brief, the Cedar Project is a cohort study involving 793 young Indigenous people who use illicit drugs in Vancouver, Prince George, and Chase, BC. Vancouver is a large city in southern BC, on the traditional territory of the Coast Salish peoples. Prince George is a mid-sized city in the northern interior of BC, on the traditional territory of Lheidli T’enneh First Nation. Chase is a rural town in south-western BC, on the traditional territory of Secwepemc First Nation. Participants were eligible if they self-identified as a descendant of the Indigenous Peoples of North America; were 14-30 years old; had smoked or injected drugs in the month before enrolment, and; provided their written informed consent. The consent process involves a thorough conversation between the participant and study interviewer to ensure that the participant fully understands the Cedar Project study rationale and the potential benefits and risks associated with being a study participant. This process is the same for all participants regardless of their age, as outlined in the BC Infants Act [30]. Since 2003, participants have returned every six months to complete interviewer-administered questionnaires and provide venous blood samples, which are tested for HIV and HCV. Honoraria are provided at each follow-up visit. This analysis included data collected between 2003-2012. Indigenous collaborators and investigators, collectively known as the Cedar Project Partnership, governed the entire research process and approved this manuscript for publication. The University of British Columbia/Providence Health Care Research Ethics Board also approved the study.

### Measures

#### Resilience

The Connor-Davidson Resilience Scale (CD-RISC) [31] was included in the longitudinal questionnaires starting in 2011. The CD-RISC is a 25-item self-administered scale designed to measure ability to cope with stress. The scale consists of five factors: 1) personal competence, high standards, and tenacity; 2) trust, tolerance, and strengthening effects of stress; 3) positive acceptance of change and secure relationships; 4) control; and 5) spiritual influences. Responses are recorded on a five-point Likert scale (*not true at all* to *true nearly all the time*). Overall scores are computed by summing all responses, with higher scores indicating greater resilience. The validity of the CD-RISC has been evaluated in multiple studies, including research involving young adults seeking treatment for anxiety related to childhood maltreatment [31], and elderly Native Americans in the United States [32]. The CD-RISC score was the time-varying outcome in this study for the analysis of resilience.

### Historical trauma

As in previous studies [9], we used two time-invariant variables as proxy measures of historical trauma. These included having at least one parent who attended residential school and ever having been taken away from biological parents and placed in foster care.

### Childhood trauma

Since 2011, Cedar Project participants have been offered the onetime option of completing the Childhood Trauma Questionnaire (CTQ) [33]. The CTQ is a widely used retrospective, self-reported 28-item inventory measuring five types of childhood maltreatment: emotional abuse, physical abuse, sexual abuse, emotional neglect, and physical neglect [33]. Responses are provided using a five-point Likert scale according to frequency of experiences (from *never true* to v*ery often true*). Due to floor and ceiling effects for individual scales, subscales scores were converted into three levels of maltreatment – none (0), low/moderate (1), and severe (2). Regression coefficients were interpreted as the mean change in the resilience score for the low/moderate levels *vs*. none and severe *vs*. none levels of maltreatment.

### Other study variables

Independent study variables for this analysis were chosen based on theoretical and empirical importance and include both time-invariant and time-varying measures. Time-invariant variables included: biological sex (male *vs*. female); study location (Prince George *vs*. Chase *vs*. Vancouver); education level (less than high school *vs*. high school graduate or more); frequency that family had lived by traditional culture (never/rarely *vs*. often/always); how often family had spoken traditional languages at home (never/rarely *vs*. often/always); and ability to speak own traditional language (no *vs*. a little bit *vs*. yes). Living by traditional culture was defined as living according to values that are inherent to customary Indigenous ways of life and taught by Elders, including humility, honesty, love, respect, loyalty, remembering where you are from, and putting family first. These variables were defined by two Indigenous Elders who are traditional knowledge keepers and members of the Cedar Project Partnership.

Time-varying variables related to the previous six-month period and included: age; relationship status (single *vs*. in a relationship); frequency of living by traditional culture (never/rarely *vs*. often/always; participating in traditional ceremonies (never/rarely *vs*. often/always); accessing alcohol or drug treatment (no *vs*. yes); accessing counselling (no *vs*. yes); trying to quit using drugs (no *vs*. yes); sleeping on the streets for three or more consecutive nights (no *vs*. yes); frequency of crack smoking (less than daily *vs*. daily or more); injection drug use (no *vs*. yes); binge drinking (no *vs*. yes); blackouts from drinking (no *vs*. yes); sex work involvement (no *vs*. yes); consistency of condom use with regular or casual sexual partners (always *vs*. not always); having a sexually transmitted infection (no *vs*. yes); having been sexually assaulted (no *vs*. yes); frequency of injecting cocaine and opiates (less than daily *vs*. daily or more); binge injection drug use (no *vs*. yes); sharing rigs (no *vs*. yes); needing help to inject drugs (no *vs*. yes); HIV and HCV serostatus; and psychological distress. Participating in traditional ceremonies included: potlatch, feast, fast, burning ceremony, washing ceremony, naming ceremony, big/smoke house, rights of passage, smudge, dances, or any other traditional Indigenous ceremony. Binge drinking and binge injection drug use were defined as having gone on runs or binges of drinking and injecting more than usual, respectively. Regular sexual partners were defined as partners with whom participants had had sexual relationships lasting three months or more, and casual sexual partners were sexual relationships lasting less than three months. Sexually transmitted infections were self-reported and may have included chlamydia, genital warts, gonorrhoea, herpes, syphilis, or others. Psychological distress was measured using the Symptom Checklist-90-R (SCL-90-R) [34]. The SCL-90-R is a 90-item self-reported symptom inventory that measures the severity of nine dimensions of psychological distress symptoms (from *not at a*ll to *extremely*). Participants’ SCL-90-R scores were transformed into a Global Severity Index, which provides an average measure for an overall degree of psychological distress.

### Study participants

Of 793 participants enrolled into the Cedar Project between 2003-2012, 446 completed baseline CD-RISC and SCL-90-R questionnaires. The sample was restricted to 191 participants who had completed the CTQ and returned for at least one additional follow-up interview to allow longitudinal analysis. No significant differences were found in sex, age, childhood trauma, or mean resilience scores for participants included in this analysis compared to those who were excluded. The overall amount of missing data for the CD-RISC items ranged from 0.06 % to 1.9 % of observations. A number of study variables also had missing data, ranging from 0.05 % to 7 % of observations. The descriptive comparison between means utilized list-wise deletion. The R software with the lme4 package that was utilized for the LME analyses uses the maximum likelihood estimation method for random missing data within the outcome variable (i.e. in this study, the mean CD-RISC score) and uses list-wise deletion for missing data within independent variables.

### Statistical analysis

T-tests identified significant differences in mean resilience scores for each dichotomous variable; robust t-tests were used when Levene’s test indicated unequal variances. One-way variance analysis was used for variables with more than two categories. Next, separate linear mixed effects (LME) models estimated the effect of each study variable on mean change in resilience scores over three follow-ups between 2011-2012. Bayesian Information Criteria was used to choose between fixed or random effect handling of study variables. Associations between study variables and resilience were tested in unadjusted analyses; those significant at *p* < 0.1 were included in multivariate models. Potential confounders specific to each model were chosen if they were associated with the study variable and mean resilience score at *p* < 0.2. Potential confounders included: sexual identity, parent attended residential school, ever been in foster care, city, relationship status, education level, and childhood maltreatment. Sex was included in every model to account for gender differences. Time-varying age was included in every model because of its potential importance as a confounder relative to the time-induced cohort effect adjustment in this study. R statistical software Version 2.15.0 with the lme4 package [35] was used [36].

## Results

Descriptive statistics of demographic variables, historical trauma, childhood maltreatment, and resilience scores are displayed in Table 1. In 2011, participants’ mean age was 28.9 years (SD: 5.1); 51 % (n = 97) were women. Fifty-three percent were based in Prince George, 39 % in Vancouver, and 8 % in Chase. Nearly half (48 %) of participants had at least one parent who had attended residential school, and most (71 %) had been in foster care.

**Table 1:** Baseline comparisons of mean resilience scores by demographic and historical trauma variables and childhood maltreatment experiences among Cedar Project participants (*n* = 191)

	Baseline frequencies		Resilience score		*p*-*value*
	*N*	%	Mean	SD	
All participants	191	100 %	62.04	22.22	-
Demographic and historical trauma variables					
Age (mean, SD)	28.89	5.07	-	-	-
Sex					
Male	94	49 %	64.12	22.37	0.871
Female	97	51 %	60.72	23.65	
Location					
Prince George	102	53 %	60.13	25.22	0.248
Chase	15	8 %	68.93	13.53	
Vancouver	74	39 %	63.75	21.71	
Any parent attended residential school					
No	41	22 %	63.97	21.45	0.629
Unsure	57	30 %	61.12	23.27	
At least one parent attended	92	48 %	62.65	23.97	
Ever in Foster Care					
No	56	29 %	68.35	19.15	0.044
Yes	135	71 %	59.99	24.07	
Education					
Less than high school	158	84 %	61.55	22.73	0.037
High school or higher	31	16 %	67.83	19.21	
Relationship status					
Single	19	10 %	64.06	15.01	0.798
In a relationship	169	90 %	62.22	23.83	
Childhood maltreatment severity					
Emotional abuse					
None	57	31 %	64.13	24.27	0.503
Low/Moderate	68	37 %	64.70	19.90	
Severe	61	33 %	58.06	25.02	
Physical abuse					
None	81	44 %	63.65	23.34	0.894
Low/Moderate	28	15 %	63.80	22.15	
Severe	77	41 %	60.52	23.26	
Sexual abuse					
None	80	43 %	62.36	24.67	0.996
Low/Moderate	33	18 %	62.57	24.67	
Severe	72	39 %	62.40	22.89	
Emotional neglect					
None	53	29 %	69.94	20.49	0.005
Low/Moderate	95	52 %	60.74	23.21	
Severe	36	20 %	53.08	23.41	
Physical neglect					
None	39	21 %	67.47	19.11	0.580
Low/Moderate	73	40 %	62.38	23.84	
Severe	72	39 %	59.53	24.00	

SD = standard deviation

Sixty-nine percent of participants reported having been emotionally abused; among whom, 33 % reported severe abuse. Fifty-six percent had been physically abused, among whom 41 % reported severe abuse. Fifty-seven percent had been sexually abused, among whom 39 % reported severe abuse. Seventy-two percent had been emotionally neglected, among whom 20 % reported severe neglect. Finally, 79 % had been physically neglected, among whom 39 % reported severe neglect.

Reliability assessments suggested that Cedar Project data had very good fit to the hypothesized model (α = 0.961). The mean resilience score was 62.04 (SD: 22.2) for all participants with no significant difference between men and women. On average, greater resilience scores were observed among participants who had never been in foster care (*p* = 0.044) and those who had graduated from high school (*p* = 0.037). Differences in mean resilience for childhood maltreatment were found only for emotional neglect, with participants reporting low/moderate or severe neglect having lower mean resilience scores than participants who reported no emotional neglect (*p* = 0.005).

### Protective factors associated with resilience

Table 2 presents results of LME models for all participants. Adjusted results are presented here. Examining the impact of time-invariant cultural factors, having a family who had often or always lived by traditional culture was associated with higher mean resilience scores (B = 7.70, *p* = 0.004). Having a family who had often or always spoken traditional languages at home was also associated with higher resilience (B = 10.52, *p* < 0.001).

**Table 2:** Unadjusted and adjusted LME models predicting the effects of study variables on mean resilience scores among Cedar Project participants (*n* = 191)

	B	SE	*t*-value	95 % CI	*p*-value	Adjusted B	SE	*t*-value	95 % CI	*p*-value
Potential Confounders										
Age	0.29	0.27	1.05	−0.25, 0.83	0.293					
Female sex	−2.59	2.77	−0.93	8.02, 2.85	0.353					
Ever in Foster Care	−2.24	3.04	−0.74	−8.2-, 3.73	0.463					
Parents attended residential school										
No	-									
Unsure	−4.44	3.86	−1.15	−12.02, 3.13	0.251					
At least one parent	1.39	3.60	0.39	−5.66, 8.44	0.700					
Location										
Prince George	-									
Chase	6.03	5.32	1.13	−4.41, 16.46	0.260					
Vancouver	−2.81	2.93	−0.96	−8.55, 2.92	0.338					
High school education or higher	7.25	3.73	1.94	−0.06, 14.55	0.053					
In a relationship	−7.00	4.27	−1.64	−15.37, 1.36	0.102					
Childhood maltreatment severity										
Emotional abuse										
None	-					-				
Low/Moderate	2.86	3.53	0.81	−4.05, 9.77	0.419	-	-	-	-	-
Severe	1.93	3.57	0.54	−5.07, 8.93	0.590	-	-	-	-	-
Physical abuse										
None	-					-				
Low/Moderate	5.60	3.77	1.48	−1.80, 12.99	0.140	-	-	-	-	-
Severe	2.06	3.13	0.66	−4.08, 8.19	0.513	-	-	-	-	-
Sexual abuse										
None	-					-				
Low/Moderate	−1.01	3.90	−0.26	−8.74, 6.53	0.796	-	-	-	-	-
Severe	2.59	3.07	0.84	−3.43, 8.61	0.400	-	-	-	-	-
Emotional neglect^a^										
None	-					-				
Low/Moderate	−5.44	3.19	−1.70	−11.69, 0.82	0.090	−5.48	3.19	−1.72	−11.73, 0.78	0.088
Severe	−12.96	4.01	−3.23	−20.83, −5.09	0.001	−13.34	4.04	−3.30	−21.25, −5.42	0.001
Physical neglect										
None	-					-				
Low/Moderate	1.35	3.85	0.35	−6.20, 8.90	0.727	-	-	-	-	-
Severe	−1.38	3.864	−0.357	−8.95, 6.20	0.721	-	-	-	-	-
Cultural connectedness										
Family often/always lived by traditional culture^b^	7.96	2.55	3.13	2.97, 12.95	0.002	7.70	2.64	2.92	2.53, 12.87	0.004
Traditional language often/always spoken at home^c^	10.66	2.41	4.43	5.94, 15.38	<0.001	10.52	2.45	4.29	5.72, 15.33	<0.001
Know how to speak traditional language^d^										
No	-					-				
A little bit	1.70	2.45	0.69	−3.09, 6.49	0.25	2.28	2.46	0.93	−2.55, 2.71	0.178
Yes	13.37	4.19	3.19	5.15, 21.58	0.001	13.06	4.19	3.12	4.85, 21.27	0.001
Often/always lived by traditional culture in past six months^e^	7.15	2.76	2.59	1.74, 12.55	0.010	6.50	2.88	2.26	0.86, 12.14	0.025
Participated in traditional ceremonies^f^	3.44	2.04	1.68	−0.56, 7.45	0.095	2.68	2.08	1.29	−1.40, 6.76	0.199
Other protective factors in the past six months										
Accessed drug/alcohol treatment^g^	3.48	2.22	1.57	−0.87, 7.82	0.118	4.84	2.29	2.11	0.35, 9.34	0.036
Accessed any counselling^h^	3.86	2.38	1.62	−0.80, 8.52	0.105	4.21	2.38	1.77	−0.46, 8.89	0.079
Tried quitting drugs^i^	4.72	2.79	1.69	−0.75, 10.19	0.092	4.98	2.85	1.75	−0.60, 10.57	0.075
Risk factors in the past six months										
Slept on streets for >3 nights	−4.55	2.99	−1.52	−10.40, 1.31	0.130	-	-	-	-	-
Daily crack smoking^j^	−5.95	2.56	−2.32	−10.97, −0.92	0.021	−5.42	2.67	−2.03	−10.66, −0.18	0.044
Injected drugs^k^	−4.41	2.65	−1.66	−9.60, 0.79	0.098	−4.12	2.75	−1.50	−9.50, 1.27	0.136
Sex work involvement	−4.15	3.11	−1.33	−10.24, 1.95	0.185	-	-	-	-	-
Did not always use condoms with casual partners	−0.36	4.136	−0.09	−8.47, 7.74	0.936	-	-	-	-	-
Did not always use condoms use with regular partners	4.48	5.10	0.88	−5.52, 14.48	0.383	-	-	-	-	-
Sexually transmitted infection	−1.16	5.04	−0.23	−11.04, 8.73	0.818	-	-	-	-	-
Sexual assault^l^	−14.61	6.96	−2.10	−28.24, −0.98	0.037	−14.42	6.97	−2.07	−28.09, −0.76	0.041
Blackouts from drinking^m^	−5.75	2.65	−2.17	−10.97, −0.56	0.032	−6.19	2.77	−2.23	−11.62, −0.75	0.027
Binge drinking	−1.54	3.11	−0.49	−7.63, 4.56	0.625	-	-	-	-	-
HIV-positive serostatus	−0.18	3.85	−0.05	−7.73, 7.36	0.960	-	-	-	-	-
HCV-positive serostatus	0.32	3.05	0.10	−6.63, 6.34	0.920	-	-	-	-	-
Psychological distress	−1.37	1.42	−0.97	−4.14, 1.40	0.333					

SD = standard deviation

^a,d,h,l^Adjusted model included confounders age and sex

^b,j,m^Adjusted model included confounders age, sex, education level, and childhood emotional neglect

^c,e,k^Adjusted model included confounders age, sex, and emotional neglect

^f,g^Adjusted model included confounders age, sex, education level, relationship status, and emotional neglect

^i^Adjusted model included confounders age, sex, education level, and having ever been in foster care

Speaking traditional languages had the strongest positive influence on participants’ resilience over time. Those who currently knew how to speak their traditional language had, on average, resilience scores that were 13.06 points higher (*p* = 0.001). Additionally, often/always living by traditional culture in the past six months was significantly associated with higher resilience scores (B = 6.50, *p* = 0.025). In the unadjusted model, participating in traditional ceremonies in the previous six months was significantly associated with an increased mean resilience score. However, the association was no longer significant after adjusting for confounders.

Having accessed drug or alcohol treatment in the past six months was also significantly associated with higher mean resilience scores (B = 4.84, *p* = 0.036). Further, although having tried to quit using drugs in the past six months was associated with higher resilience in the unadjusted model (B = 4.72, *p* = 0.092), this association was only marginally significant after adjusting for confounders (B = 4.98, *p* = 0.075).

### Risk factors associated with resilience

Of the five types of childhood maltreatment, only emotional neglect was associated with mean resilience score, with participants who had experienced severe emotional neglect having significantly lower mean resilience scores (B = −13.33, *p* = 0.001).

For the time-varying risk factors, having been sexually assaulted had the greatest negative effect on participants’ resilience. Participants who reported sexual assault had, on average, mean resilience scores that were −14.42 lower (*p* = 0.041). In addition, smoking crack daily (B = −5.42, *p* = 0.044) and having had blackouts from drinking alcohol were both significantly associated with diminished mean resilience scores-(B = −6.19, *p* = 0.027). Though there was a marginal association between having injected drugs and lower mean resilience in unadjusted analysis, adjusting for confounders attenuated the result.

## Discussion

Indigenous scholars emphasize that resilience is inherent to Indigenous cultures and that strength based in culture makes a vital contribution to the health of Indigenous peoples today [37]. The tenacity and strength of Indigenous peoples has been demonstrated in 500 years of resistance against colonial efforts to suppress their culture and self-determination. As this study has demonstrated, young Indigenous people who use drugs face considerable challenges to their resilience. A large proportion faced substantial adversities in their early lives, including having a parent who may have struggled with the effects of residential school, experiencing childhood abuse and/or neglect, and having been in foster care. Associations between drug- and sex-related HIV and HCV risk factors and decreased resilience are of great concern. At the same time, it is profoundly reassuring that participants who had access to the buffers of culture and language exhibited increased resilience. Quitting drugs and seeking help were also positively associated with resilience. These findings have critically important implications for public health programming to support the strengths of young Indigenous people who use drugs in Canada.

### Comparing resilience scores with other studies

Drawing inferences when comparing resilience scores with other samples is difficult, as resilience is dynamic and influenced by many intersecting factors. However, baseline mean resilience scores for Cedar Project participants were similar to other Canadian studies including a sample of street-involved youth [38] and an ethnically diverse sample of young urban people transitioning out of the child welfare system [39].

### Culture and resilience

Factors that reflected familial connection to culture, including having a family who often/always lived by traditional culture and often/always spoke their traditional language at home, were both very strong predictors of participants’ current level of resilience. These findings reflect the intergenerational strength of familial cultural resources, as they provided an ongoing protective effect that enhanced participants’ ability to cope with stress later in life – regardless of childhood maltreatment. These results are also consistent with research that emphasized Indigenous women’s sources of “inner strength” (p. 87) that had originated from cultural resources within their kinship systems and with the natural and spiritual worlds [40]. In addition, these findings highlight the importance of funding health interventions that support Indigenous families in urban centres to connect to culture, languages, and spirituality, which create opportunities to nurture cultural pride and connection to community in children and youth.

Participants who were currently often/always living by traditional culture had significantly higher mean resilience scores over the study period. These findings also resonate with previous research that measured the degree to which living by traditional culture provides a buffering effect on young Indigenous peoples’ mental and emotional health [41]. Importantly, our study demonstrates the protective effect of culture and cultural identity on stress-coping ability among young people who live with the complex challenges of being street-involved, dependent on substances, and facing everyday stresses of structural and interpersonal violence. In addition, knowledge of their traditional language had the strongest positive effects on participants’ mean resilience scores. Traditional languages are considered fundamental components of Indigenous cultures [42]. It is likely that the participants in this study who knew how to speak their traditional language also had strong cultural identities and could therefore connect to the values, concepts, and beliefs that are embedded in language. What is more, the enduring health benefits of knowing their traditional language were evident regardless of any history of historical or lifetime trauma.

It is worth noting that although participating in traditional ceremonies in the past six months did not reach statistical significance, the mean scores of participants who had done so were significantly higher than the mean scores of those who had not in the descriptive analysis. This finding is consistent with other studies that have found that young Indigenous peoples’ participation in traditional activities protects against adverse mental health outcomes and harmful ways of coping with stress, such as substance use [41]. Recent participation in traditional ceremonies may have lacked significance in the longitudinal analysis because young Indigenous people in cities do not have consistent access to traditional activities. This may be explained by the fact that most traditional protocols require abstinence for participation in sacred ceremonies. Our study findings support calls for innovative programming to provide young Indigenous people who use drugs with culturally acceptable opportunities to access traditional activities, languages, and teachings that promote positive stress-coping [42].

This study also demonstrated a strong association between having accessed alcohol or drug treatment and increased mean resilience scores. In addition, we found marginal adjusted associations between increased resilience scores and having accessed counselling and having tried to quit using drugs. Because of the dynamic nature of resilience, seeking therapeutic help for substance use and attempting to quit drugs may be a demonstration of participants’ resilience; likewise, these behaviours may help to reinforce or promote resilience. These findings are consistent with previous research involving Indigenous adults living in Edmonton, Canada, which demonstrated that higher scores on an Indigenous enculturation scale were associated with decreased illicit drug use [43]. Taken together, these results suggest that incorporating cultural teachings, values, and traditional healing within primary health care and therapeutic health services may be especially beneficial in supporting wellness among young Indigenous people who use drugs. Indeed, such wellness-focused interventions may facilitate healing from the effects of intergenerational trauma, and support resilience against experiences of structural and interpersonal violence. Compared to public health efforts that focus solely on addressing risk factors, interventions that cultivate cultural resilience may also reduce or prevent young Indigenous people’s susceptibility to HIV and HCV exposure within high-risk environments.

### Challenges to resilience

It is deeply concerning that 92 % of participants in this study had experienced some type of maltreatment (data not shown) and that high proportions experienced severe maltreatment. However, only emotional neglect was significantly associated with decreased mean resilience scores. Emotional neglect is often characterized by acts of omission by caregivers who persistently deprive children of basic psychological and emotional nurturing, encouragement, and feelings of belonging [44]. Effects of childhood emotional neglect can extend into adulthood and increase the likelihood that adults will experience diminished cognitive, social, and emotional functioning, and most notably, have difficulties with positive adaptation and stress-coping [44, 45]. Recently, the Truth and Reconciliation Commission of Canada described the egregious negligence of generations of Indigenous children in residential schools as “institutionalized child neglect” [46]. Residential school survivors have recalled feeling isolated, deprived of love, nurturing, or comfort, and being instilled with a sense of worthlessness. This approach was highly dissimilar to traditional parenting styles and impacted survivors’ own parenting. Consequent intergenerational trauma may be affecting young Indigenous people today who turn to drugs and alcohol in the absence of safe and effective coping mechanisms. Mental and public health interventions serving young Indigenous people who use drugs must be cognizant of possible intergenerational impacts of emotional neglect and its specific effect on resilience.

Descriptive findings in this study demonstrated that participants who had been in foster care had significantly lower mean resilience scores than participants who had not. Despite not retaining significance in adjusted models, this finding merits attention as Indigenous leaders have called for an end to the cycle of child apprehension through long-term, culturally-relevant resources to support young people to heal and recover the resilience innate to their cultures and ways of knowing [7]. Future research must involve young Indigenous people who use drugs and who have experienced the child welfare system to identify how to support healthy attachments to their families and cultures [47].

Sexual assault had the strongest negative association with participants’ mean resilience scores in this study. This finding is concerning, as approximately half of those who experience sexual assault develop post-traumatic stress disorder symptoms if unable to access timely interventions that facilitate positive stress-coping and adaptation [48]. Survivors of sexual assault who suffer from post-traumatic stress disorder are more likely to self-blame and engage in harmful coping strategies such as heavy alcohol and drug use, thereby increasing their vulnerability for HIV and HCV infection [49]. Previous research has highlighted the relationship between intergenerational trauma caused by the residential school system and sexual violence experienced by young Indigenous women who use drugs [14]. Sexual assault prevention and intervention strategies for young Indigenous people who use drugs must therefore be trauma-informed and specifically tailored to establish trust-based relationships within culturally-safe settings.

Smoking crack daily and blackout drinking were also independently associated with decreased mean resilience scores. Few studies have explored the association between resilience and problematic substance use among vulnerable populations [38, 50]. However, research has suggested that poor mental health–especially depression and post–traumatic stress disorder–precedes heavy alcohol consumption and cocaine use (rather than vice versa) [51, 52]. These findings may be interpreted as indicating that young Indigenous people use drugs to cope with stress because of a lack of alternative coping skills or access to culturally-relevant mental health and addiction resources within urban centres. This potentiality is deeply concerning given that HIV risk and infection has been linked with unsafe sex practices that often coincide with heavy alcohol consumption and crack smoking [53, 54].

There are several important limitations to this study. First, it utilizes self-reported behavioural data obtained from a non-probabilistic sample. Although we cannot rule out selection bias, we are confident that our recruitment methods and rigorous eligibility criteria ensured that our sample is representative of Indigenous young people who use drugs in Vancouver, Prince George, and Chase. There was potential for recall bias, socially desirable reporting, and misclassification of exposures (except for HIV/HCV serostatus) and the outcome variable. Additionally, we cannot draw conclusions regarding the causality between the time-varying study variables and resilience. We also acknowledge that the CD-RISC is based on Eurocentric concepts and is likely unable to capture some of the deeper sociocultural and ecological factors that contribute to the resilience of Cedar Project participants [25].

## Conclusions

In conclusion, this study has demonstrated what many Indigenous scholars and Elders have known for generations: that cultural teachings, values, and languages are the foundations of resilience among Indigenous peoples. In the aftermath of colonization, these foundations continue to function as “cultural buffers” [55] that protect Indigenous peoples from severe health outcomes, including HIV and HCV infection. The young Indigenous people in this study are survivors, as they have adapted to and lived through multiple and intersecting adversities. This study has demonstrated that those young people who had access to culture and languages were buffered both psychologically and emotionally. Conversely, this study underscored the importance of culturally-safe and trauma-informed interventions that prevent any further decline in the strength of young people–especially those who have experienced sexual violence and those using alcohol and drugs very heavily. Supporting the reconstruction of cultural identities among young Indigenous people living in urban centres who are either disconnected from their cultures or have never experienced their cultures may be challenging [26, 27]. Young Indigenous people who use drugs must be involved in the design, implementation, and evaluation of any programs or resources that intend to support cultural identity, cultural pride, and cultural resilience.

## Abbreviations

BC: British Columbia
CTQ: Childhood Trauma Questionnaire
CD-RISC: Connor Davidson Resilience Scale
HCV: Hepatitis C virus
HIV: Human Immunodeficiency Virus
SCL-90-R: Symptom Checklist 90-Revised
